# Supplementary material for: Hollow Spherical Pd/CdS/NiS with Carrier Spatial Separation for Photocatalytic Hydrogen Generation
Source: Nanomaterials (Basel). 2023 Apr 10;13(8):1326. doi: 10.3390/nano13081326 (PMC10143208; doi:10.3390/nano13081326)
Supplement: Supplementary file 1 [file nanomaterials-13-01326-s001.zip › nanomaterials-2312462-supplementary.pdf]

nanomaterials

*Supplementary Material*

# Hollow Spherical Pd/CdS/NiS with Carrier Spatial Separation for Photocatalytic Hydrogen Generation

**Xiao Wang [1,2], Fei Zhao [1,2], Nan Zhang [1,2], Wenli Wu [1,2] and Yuhua Wang [1,2,*]**

[1] School of Materials and Energy, Lanzhou University, Lanzhou 730000, China; wangxiao2020@lzu.edu.cn (X.W.); zhaof2016@lzu.edu.cn (F.Z.); zhangn2018@lzu.edu.cn (N.Z.); wuwl2021@lzu.edu.cn (W.W.)
[2] National and Local Joint Engineering Laboratory for Optical Conversion Materials and Technology, Lanzhou University, Lanzhou 730000, China
* Correspondence: wyh@lzu.edu.cn; Tel.: +86-931-8912772; Fax: +86-931-8913554

**Table S1.** ICP-OES test results of Pd/CdS/NiS samples

| Elements | Concentration (mg/L) | Weight Ratio (calculated by CdS, NiS and Pd) |
|---|---|---|
| Cd | 34.07 | 1 |
| Ni | 0.6636 | 0.0233 |
| Pd | 0.2791 | 0.00635 |

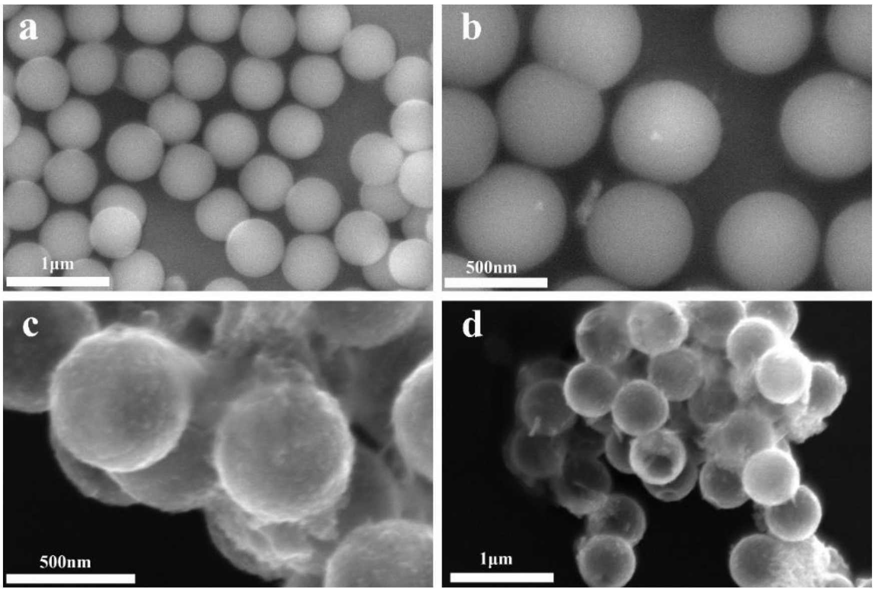


**Figure S1.** SEM figures of (**a**) $SiO_2$; (**b**) $SiO_2$@Pd; (**c**) $SiO_2$@ Pd/CdS/NiS; (**d**) hollow Pd/CdS/NiS

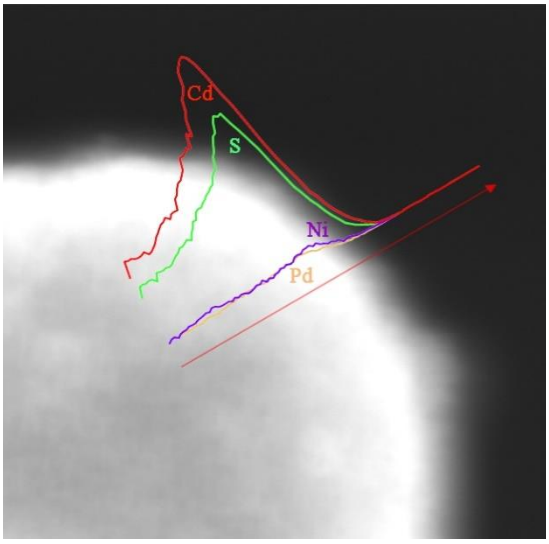


**Figure S2.** HAADF TEM image and the corresponding EDS line-scan of Pd/CdS/NiS

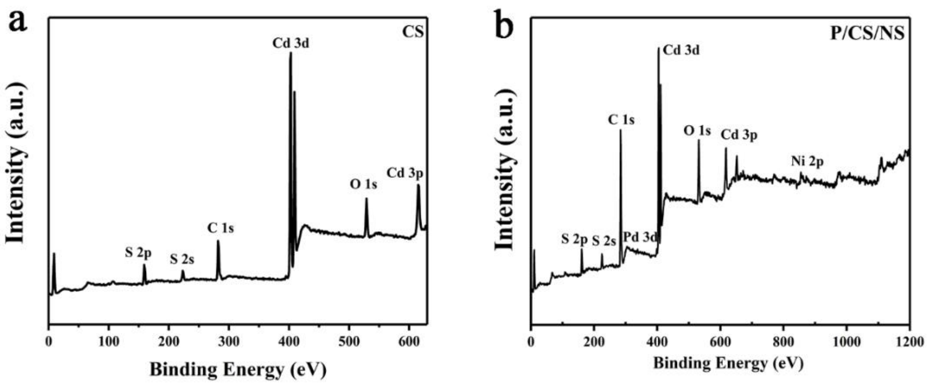


**Figure S3.** Fully scanned XPS spectra of (**a**) CdS; (**b**) Pd/CdS/NiS

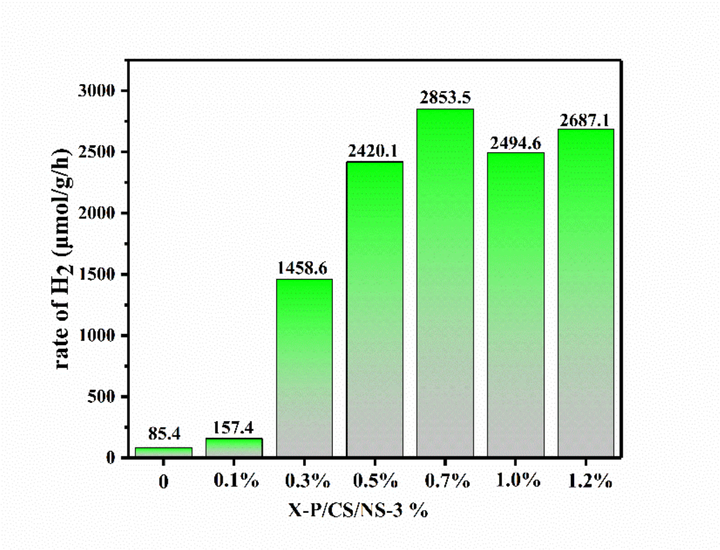


**Figure S4.** $H_2$ production in Pd/CdS/NiS loaded with different ratios of Pd per unit time (λ≥ 420 nm)

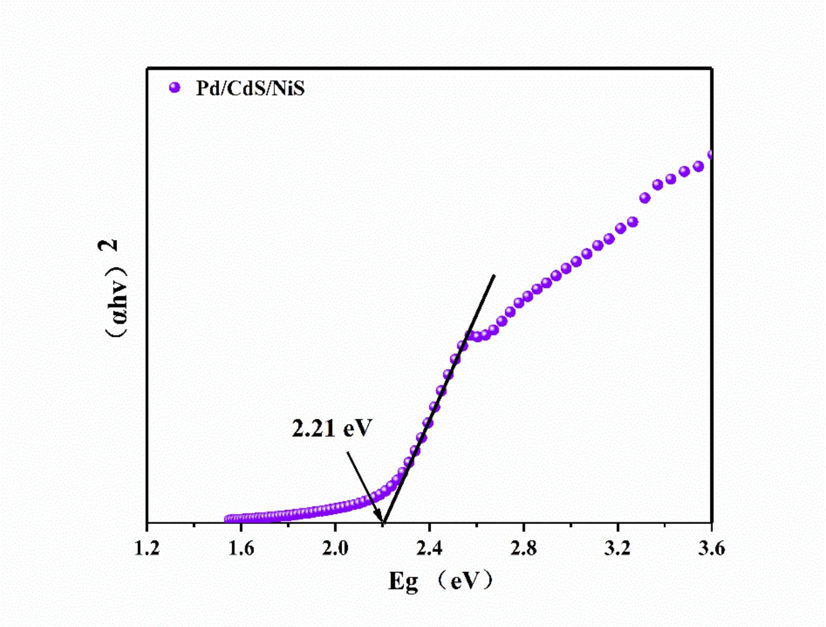


**Figure S5.** Tauc plots of Pd/CdS/NiS

**Table S2.** Comparison of photocatalytic $H_2$ generation performance.

| Catalyst | Sacrificial agents | Light source | $H_2$ evolution rate (μmol/h/g) | Improve times | Ref. |
|---|---|---|---|---|---|
| Pd/CdS/NiS | 0.1 M $Na_2SO_3$ and $Na_2S$ | 300 W Xenon lamp (420–800 nm) | 3814 | 33.4 | This work |
| CdS/UiO-66(10) | 20% lactic acid | 300 W Xenon lamp (λ > 380 nm) | 1725 | 5.5 | [1] |
| $Ni_3S_4$/CdS | 0.35 M $Na_2S$, and 0.25 M $Na_2SO_3$ | 300 W Xenon lamp (420–800 nm) | 5526 | 21 | [2] |
| $TiO_2$/Au/CdS | 30% lactic acid | 300 W Xenon lamp (420–800 nm) | 4600 | 3.2 | [3] |
| CdS/NiS-PBA | 0.35 M Na2S, and 0.25 M Na2SO3 | 300 W Xenon lamp (420–800 nm) | 8874 | 13.9 | [4] |
| CdS/Mo(0.38)-VC | 10% lactic acid | 300 W Xenon lamp (420–800 nm) | 2267 | 4.4 | [5] |
| $PtP_2$@C/CdS | 0.35 M $Na_2S$, and 0.25 M $Na_2SO_3$ | 3 W 420 nm LED lamps | 9760 | 34.8 | [6] |
| CdS/np-rGO | 0.35 M $Na_2S$, and 0.25 M $Na_2SO_3$ | 300 W Xenon lamp (420–700 nm) | 2127 | 3.6 | [7] |
| NC@Co-NCT/CdS | 30% lactic acid | 300 W Xenon lamp (420–800 nm) | 3800 | 5.8 | [8] |

| | | | | | |
|---|---|---|---|---|---|
| CdS-fullerene/graphene | 25% methanol | 300 W Xenon lamp (420–800 nm) | 127.1 | 4.3 | [9] |

## References


1. Xu, H.-Q.; Yang, S.; Ma, X.; Huang, J.; Jiang, H.-L. Unveiling Charge-Separation Dynamics in CdS/Metal–Organic Framework Composites for Enhanced Photocatalysis, *ACS Catalysis*, **2018**, *8* 11615–11621.
2. Wang, Y.; Zhao, J.; Hou, W.; Xu, Y. Decoration of CdS nanowires with Ni3S4 nanoballs enhancing H2 and H2O2 production under visible light, *Applied Catalysis B: Environmental*, **2022**, *310* 121350.
3. Wang, W.; Zhang, Y.; Xie, J.; Wang, Y.; Cao, S.; Ping, H.; Zou, Z.; Zeng, H.; Wang, W.; Fu, Z. Bioinspired Strategy for Efficient TiO(2)/Au/CdS Photocatalysts Based On Mesocrystal Superstructures in Biominerals and Charge-Transfer Pathway in Natural Photosynthesis, *ACS Appl Mater Interfaces*, **2023**, *15* 2996–3005.
4. Meng, X.; Wang, S.; Zhang, C.; Dong, C.; Li, R.; Li, B.; Wang, Q.; Ding, Y. Boosting Hydrogen Evolution Performance of a CdS-Based Photocatalyst: In Situ Transition from Type I to Type II Heterojunction during Photocatalysis, *ACS Catalysis*, **2022**, *12* 10115–10126.
5. Lei, Y.; Ng, K.H.; Zhu, Y.; Zhang, Y.; Li, Z.; Xu, S.; Huang, J.; Hu, J.; Chen, Z.; Cai, W.; Lai, Y. Mo-activated VC as effective cocatalyst for an enhanced photocatalytic hydrogen evolution activity of CdS, *Chemical Engineering Journal*, **2023**, *452* 139325.
6. Xu, J.; Zhong, W.; Gao, D.; Wang, X.; Wang, P.; Yu, H. Phosphorus-enriched platinum diphosphide nanodots as a highly efficient cocatalyst for photocatalytic H2 evolution of CdS, *Chemical Engineering Journal*, **2022**, *439* 135758.
7. Yuan, C.; Lv, H.; Zhang, Y.; Fei, Q.; Xiao, D.; Yin, H.; Lu, Z.; Zhang, Y. Three-dimensional nanoporous heterojunction of CdS/np-rGO for highly efficient photocatalytic hydrogen evolution under visible light, *Carbon*, **2023**, *206* 237–245.
8. Li, X.; Song, S.; Gao, Y.; Ge, L.; Song,W.; Ma, T.; Liu, J. Identification of the Charge Transfer Channel in Cobalt Encapsulated Hollow Nitrogen-Doped Carbon Matrix@CdS Heterostructure for Photocatalytic Hydrogen Evolution, *Small*, **2021**, *17* e2101315.
9. Wang, W.; Tao, Y.; Fan, J.; Yan, Z.; Shang, H.; Phillips, D.L.; Chen, M.; Li, G. Fullerene–Graphene Acceptor Drives Ultrafast Carrier Dynamics for Sustainable CdS Photocatalytic Hydrogen Evolution, *Advanced Functional Materials*, **2022**, *32* 2201357.
